# Partial response after transcatheter arterial infusion chemotherapy in a patient with systemic chemotherapy-resistant unresectable colon cancer and hepatic metastasis: (case report)

**DOI:** 10.1186/1477-7819-11-203

**Published:** 2013-08-17

**Authors:** Katsuji Sawai, Takanori Goi, Kenji Koneri, Kanji Katayama, Akio Yamaguchi

**Affiliations:** 1 First Department of Surgery, University of Fukui, 23-3, Eiheiji-cho, Yoshida-gun, Fukui, Japan

**Keywords:** Hepatic arterial infusion chemotherapy, Resistance to systemic chemotherapy, Unresectable colon cancer

## Abstract

We report here a case of partial response to hepatic arterial infusion chemotherapy in a patient who developed serious hepatic failure due to unresectable colorectal cancer and hepatic metastasis and showed resistance to systemic chemotherapy with molecular targeted drugs, mFOLFOX6, and FOLFIRI. The patient was a 60-year-old woman who underwent sigmoidectomy for sigmoid colon cancer, lateral posterior hepatic segmentectomy for metastatic liver cancer, and postoperative radiation therapy for metastatic lung cancer. As first-line systemic chemotherapy, mFOLFOX6 (oxaliplatin, 5-fluorouracil, and leucovorin), bevacizumab + FOLFIRI (irinotecan, 5-fluorouracil, leucovorin), and anti-epidermal growth factor receptor antibody + irinotecan were administered, in that order. However, recurrent hepatic metastasis was exacerbated, which induced serious hepatic failure manifested by general malaise, jaundice, abnormal hepatic function, difficulty in walking due to bilateral lower extremity edema, and decreased appetite. The patient was admitted in a serious condition. After hospitalization, the patient received hepatic arterial infusion chemotherapy with 5-fluorouracil and l-leucovorin. After two complete courses, the symptoms improved. The patient’s performance status also improved, and she was discharged from the hospital. Four months after discharge, the patient had continued outpatient chemotherapy and maintained excellent performance status. Although HAIC is not presently considered an alternative to systemic chemotherapy, it is sometimes effective in patients who show resistance to molecular targeted drug therapy, FOLFOX, and FOLFIRI, and in whom hepatic metastasis is a key factor in determining prognosis and serious hepatic failure. Further studies should be performed in the future to verify these findings.

## Background

The Japanese guidelines for colorectal cancer treatment and the National Comprehensive Cancer Network (NCCN) Clinical Practice Guidelines in Oncology for Colon and Rectal Cancers recommend systemic administration of l-leucovorin, 5-fluorouracil, irinotecan, oxaliplatin, bevacizumab, and anti-epidermal growth factor receptor (EGFR) antibody for unresectable or recurrent colon cancer [1]. However, there is no effective therapy for patients who have developed resistance to this therapy. We report here a case of colorectal cancer with hepatic metastasis, which was resistant to systemic therapy, and serious hepatic failure, for which hepatic arterial infusion chemotherapy (HAIC) was effective.

## Case presentation

The patient was a 60-year-old woman with chief complaints of general malaise, jaundice, bilateral lower extremity edema, and decreased appetite. At admission, she presented with conjunctival anemia and jaundice, mild tenderness in the right upper abdomen, a palpable liver 3 cm below the right costal margin, and bilateral lower extremity edema. Her body temperature was 37.8°C, and her performance status was 3 [2]. Routine admission serum chemistry showed a white blood cell count of 17,200/μl (normal range = 3400 to 9600/μl), and the following concentrations: hemoglobin, 8.1 g/dl (normal range = 13.4 to 17.6 g/dl); total bilirubin, 9.5 mg/dl (normal range = 0.2 to 1.2 mg/dl); aspartate transaminase, 105 IU/l (normal range = 13 to 34 IU/l), γ-glutamyl transpeptidase, 643 IU/l (normal range = 12 to 60 IU/l), lactate dehydrogenase, 1,414 IU/l (normal range = 119 to 214 IU/l), and alkaline phosphatase, 4,558 IU/l (normal range = 107 to 340 IU/l). These results indicated a hepatic function disorder. The concentrations of tumor markers CEA and CA19-9 were significantly increased to 197 ng/ml (normal range = 0 to 5.0 ng/ml) and 42.9 U/ml (normal range = 0 to 37 U/ml) respectively.

### Illness and course

Sigmoidectomy, lateral and posterior hepatic segmentectomy, and postoperative radiation therapy were performed in February 2008 to treat sigmoid colon cancer, metastatic liver cancer, and metastatic lung cancer, respectively. The final diagnosis was of sigmoid colon cancer (6 × 4 cm), type 2, invasion depth SE, lymph node metastasis N2, hepatic metastasis H2, P0, M1 stage IV [3].

**Figure 1:**
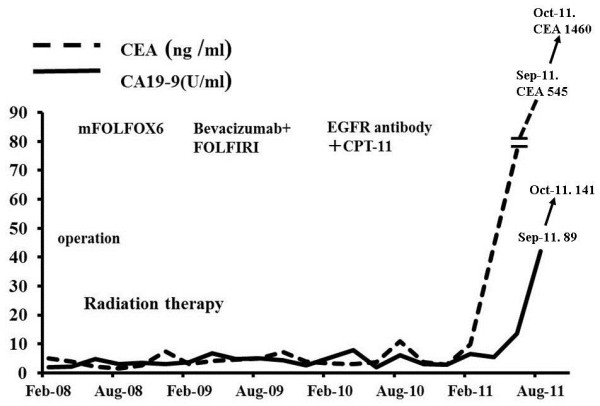
**Illness and course.** Sigmoidectomy, lateral posterior hepatic segmentectomy, and postoperative radiation therapy were performed in February 2008 for the treatment of sigmoid colon cancer, metastatic liver cancer, and metastatic lung cancer, respectively. CPT-11, irinotecan; FOFIRI, irinotecan, 5-fluorouracil, leucovorin; mFOLFOX6, oxaliplatin, 5-fluorouracil, and leucovorin.

After surgery, starting in March 2008, 12 cycles of mFOLFOX6 were administered as first-line chemotherapy, according to the Japanese guidelines for unresectable advanced colorectal cancer [4]. Follow-up computed tomography in December 2008 showed a new unresectable hepatic metastasis. Therefore, this therapy was replaced with bevacizumab + FOLFIRI as second-line treatment and 12 cycles were given. Follow-up computed tomography in December 2009 showed that the hepatic metastasis was poorly differentiated. Therefore, anti-EGFR antibody with irinotecan was administered as third-line treatment. However, the recurrent hepatic metastasis was exacerbated, and the patient developed serious hepatic failure manifested by general malaise, jaundice, abnormal hepatic function, difficulty in walking due to bilateral lower extremity edema, and decreased appetite. The patient was hospitalized in August 2011 (Figure 1).

### Clinical course

**Figure 2:**
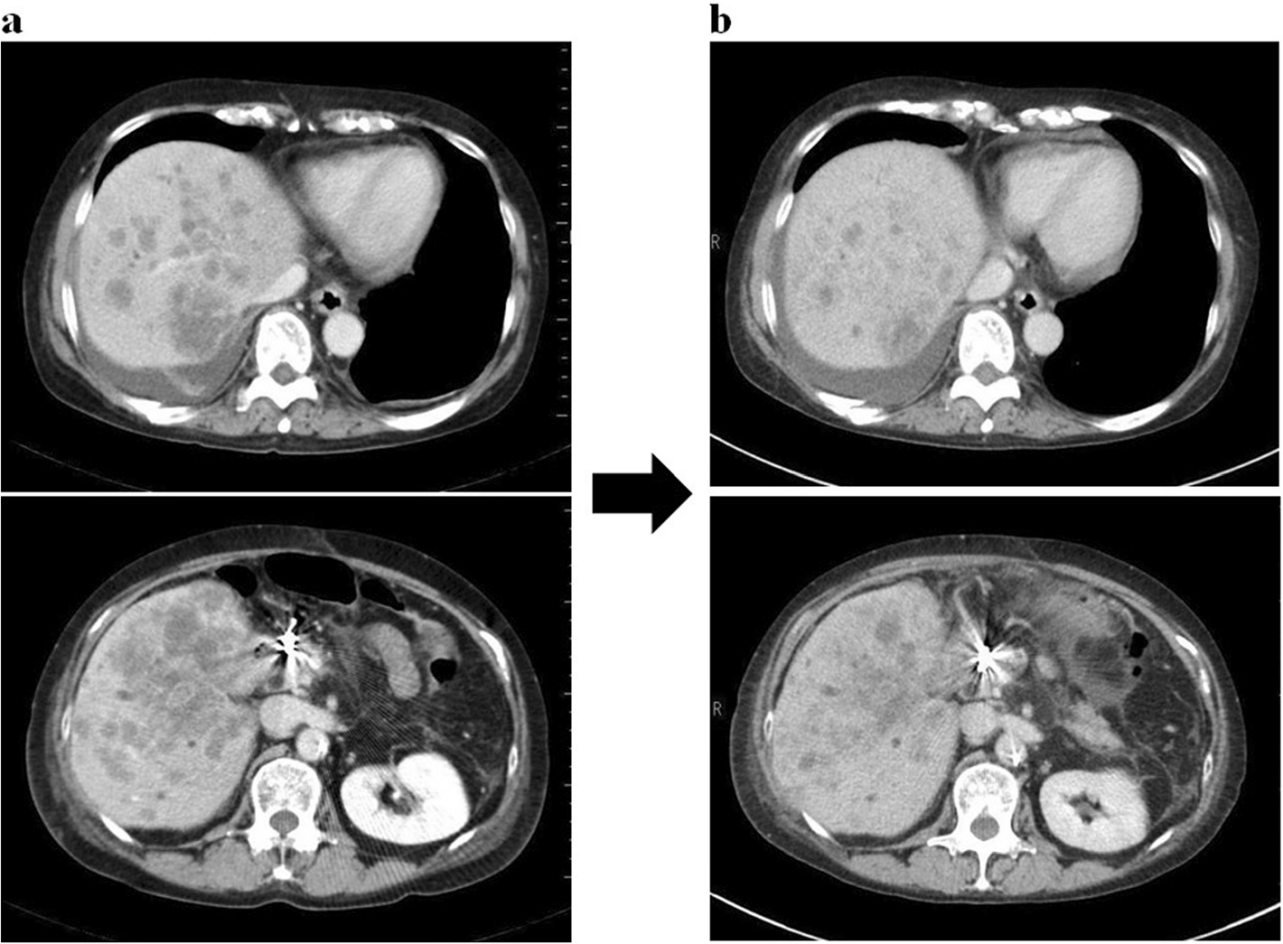
**Abdominal computed tomography. (a)** Before treatment. Metastatic lesions in both lobes of the liver were revealed. **(b)** After two courses of treatment, hepatic metastasis was decreased: The metastatic lesion in the liver had reduced, and the other lesions also tended to be reduced in size.

**Figure 3:**
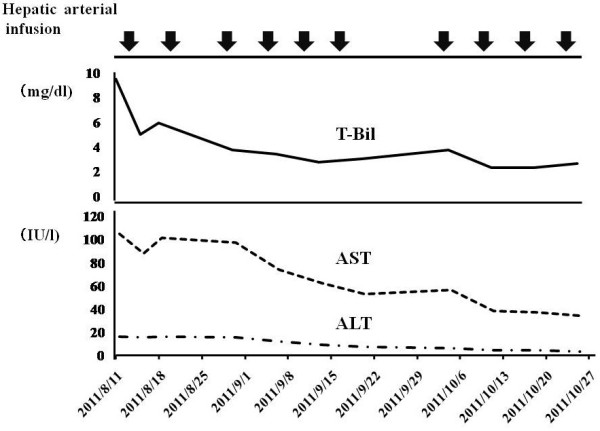
**Blood test results.** Abnormal hepatic function had decreased. ALT, alanine transaminase; AST, aspartate transaminase; T-Bil, total bilirubin.

The patient showed resistance to systemic administration of the five types of chemotherapeutic agent recommended by the Japanese guidelines for colorectal cancer treatment and the NCCN clinical practice guidelines in oncology for colon and rectal cancers [1,5]. The growth of the hepatic lesion and the abnormal hepatic function suggested that the patient had developed serious hepatic failure. Lung metastasis was also observed; however, this did not seem to affect prognosis. We explained to the patient and her family members that her condition would not be life threatening, regardless of the multiple metastases to other organs; however, the standard therapy was not indicated. We also explained the possibility of discontinuing the chemotherapy, and administering palliative care and HAIC to control the hepatic metastasis. After obtaining informed consent, HAIC was selected and performed. After altering blood flow in the gastroduodenal artery and right gastric artery during angiography, the tip of the catheter was placed in the hepatic artery, and one course of 5-fluorouracil (250 mg/m^2^) and l-leucovorin (600 mg/m^2^) was administered once a week. A once-weekly infusion for 6 weeks was defined as one course. No adverse reactions were observed during HAIC. After two courses, computed tomography showed decreased hepatic metastasis (Figure 2). According to the blood test results, abnormal hepatic function had also decreased (Figure 3). However, the levels of tumor markers CEA and CA19-9 were not decreased (Figure 1). On admission, the patient’s performance status was 3 [2] because she had difficulty in walking due to bilateral lower extremity edema and the inability to eat. After two courses of this treatment had been completed, the lower extremity edema disappeared, and the patient could eat and perform household chores well. Her performance status was 0, and she was discharged. The aforementioned conditions have been maintained for 4 months. However, 5 months later, the recurrent hepatic metastasis was exacerbated, and the patient died of serious hepatic failure. Lung metastasis was slightly increased; however, it did not seem to affect prognosis.

### Discussion

The Japanese guidelines for colorectal cancer treatment and the NCCN clinical practice guidelines in oncology for colon and rectal cancers recommend FOLFOX as the optimal treatment for patients with unresectable recurrent colon cancer with hepatic metastasis, and systemic chemotherapy with a combination of FOLFIRI and anti-VEGF antibody or anti-EGFR antibody. The median survival of patients with unresectable or advanced recurrent colon cancer who do not undergo chemotherapy has been reported as approximately 8 months. However, owing to recent developments in chemotherapy, median survival has been extended to approximately 2 years [1,5]. At the initial examination, the patient had stage IV colorectal cancer with hepatic metastasis and lung metastasis, and had a risk of digestive tract ileus due to the primary focus. Therefore, the primary focus and the hepatic metastasis were removed. Radiotherapy was performed to treat the single lung metastasis. At that time, all lesions disappeared on the images. However, 11 months after the operation, unresectable recurrent hepatic metastasis developed, and systemic chemotherapy was performed in accordance with the clinical practice guidelines for colon and rectal cancers. The patient’s condition was maintained for 2 years and 8 months after the therapy; however, she then developed serious hepatic failure manifested by abnormal hepatic function, decreased appetite, general malaise, and difficulty in walking due to bilateral lower extremity edema due to exacerbation of hepatic metastasis. Discontinuation of the chemotherapy was considered, since the standard therapy was not indicated, but the patient’s condition would not be life threatening, regardless of multiple metastases to other organs. Control of hepatic metastasis could prolong her life. There are no reports of the use of HAIC to treat patients showing resistance to systemic chemotherapy with anti-EGFR antibody, anti-EGFR antibody, mFOLFOX6, and FOLFIRI. However, some reports have shown that HAIC was effective for size regression and local control of hepatic metastasis [6,7]. Therefore, HAIC was performed after obtaining informed consent from the patient and her family members. No adverse reactions occurred, and serious hepatic failure was improved. After the patient was discharged, her quality of life improved and she could do household chores, while currently receiving outpatient treatment. Therefore, HAIC was effective in this case. Recently, some studies have reported that HAIC or a combination of HAIC and systemic chemotherapy was effective for patients who showed resistance to systemic chemotherapy [8-11]. HAIC has again received attention in patients in whom hepatic metastasis is a key factor determining prognosis. In recent years, systemic chemotherapy has seen remarkable progress.

## Conclusions

Although HAIC is not presently considered an alternative to systemic chemotherapy, it is sometimes effective in patients who show resistance to molecular targeted drug therapy, FOLFOX, and FOLFIRI, and in whom hepatic metastasis is a key factor in determining prognosis and serious hepatic failure. Further studies should be performed in the future to verify these findings.

## Consent

Written informed consent was obtained from the patient for publication of this case report and any accompanying images. A copy of the written consent is available for review by the Editor-in-Chief of this journal.

## Abbreviations

EGFR: Epidermal growth factor receptor; FOLFIRI: Irinotecan, 5-fluorouracil, leucovorin; HAIC: Hepatic arterial infusion chemotherapy; mFOLFOX6: Oxaliplatin, 5-fluorouracil, and leucovorin; NCCN: National comprehensive cancer network.

## Competing interests

The authors declare that they have no competing interests.

## Authors’ contributions

All authors read and approved the final manuscript.
